# Preparation and Characterization of Highly Ordered Mercapto-Modified Bridged Silsesquioxane for Removing Ammonia-Nitrogen from Water

**DOI:** 10.3390/polym10080819

**Published:** 2018-07-25

**Authors:** Derong Lin, Yichen Huang, Yuanmeng Yang, Xiaomei Long, Wen Qin, Hong Chen, Qing Zhang, Zhijun Wu, Suqing Li, Dingtao Wu, Lijiang Hu, Xingwen Zhang

**Affiliations:** 1 College of Food Science, Sichuan Agricultural University, Ya’an 625014, China; 18723091689@163.com (Y.H.); sicau_yym@126.com (Y.Y.); 18428301943@163.com (X.L.); qinwen@sicau.edu.cn (W.Q.); chenhong945@sicau.edu.cn (H.C.); zhangqing@sicau.edu.cn (Q.Z.); lsq03_2001@163.com (S.L.); DT_Wu@sicau.edu.cn (D.W.); 2 School of Mechanical and Electrical Engineering, Sichuan Agricultural University, Ya’an 625014, China; wzj@sicau.edu.cn; 3 State Key Laboratory of Urban Water Resource and Environment, Harbin Institute of Technology, Harbin 150090, China; hulijiang2008@126.com (L.H.); zhangxinwen@hit.edu.cn (X.Z.)

**Keywords:** direct synthesis, grafting synthesis, mercapto-modified, bridged silsesquioxane

## Abstract

In acidic conditions, mesoporous molecular sieves SBA-15 and SBA-15-SH were synthesized. Structural characterization was carried out by powder X-ray diffraction (XRD), Fourier Transform infrared spectroscopy (FTIR), scanning electron microscopy (SEM), transmission electron microscopy (TEM), ^13^C CP MAS-NMR, ^29^Si CP MAS-NMR and nitrogen adsorption–desorption (BET). The results showed that in SBA-15-SH, the direct synthesis method made the absorption peak intensity weaker than that of SBA-15, while the post-grafted peak intensity did not change. Their spectra were different due to the C-H stretching bands of Si-O-Si and propyl groups. But their structure was still evenly distributed and was still hexangular mesoporous structure. Their pore size increased, and the H-SBA-15-SH had larger pore size. The adsorption of ammonia-nitrogen by molecular sieve was affected by the relative pressure and the concentration of ammonia-nitrogen, in which the adsorption capacity of G-SBA-15-SH was the largest and the adsorption capacity of SBA-15 was the smallest.

## 1. Introduction

Organic pollutants have gradually increased and have serious environmental impact. Adsorption has become one of the methods of wastewater treatment. Among these, the bridge group and modified silsesquioxane (SSO) have used a variety of optimization methods to promote the development and application of this mesoporous three-dimensional material because of its unique physical and chemical properties [1,2,3,4,5,6,7,8]. For example, the SSO membrane can be used for the removal of anionic compounds, and it can be easily separated from the treated medium [9]. The SSO with a cage structure is called polyhedral oligomeric silsesquioxane (POSS), and POSS was used as a new type of organic-inorganic hybrid material [10]. It was not easy to prepare under alkaline or other harsh conditions. The structure and functional integrity of the POSS nucleus can be not damaged by the “click” reaction of the new method. This was because the effective condensation reaction between the amino oxygen group and aldehyde or ketone produces oxime bonds in order to produce chemical selectivity in the subsequent reaction [11]. The two hydrophilic carboxylic acid functionalized POSS heads and two hydrophobic polystyrene trails which were covalently linked through the rigid septum can synthesize the giant Gemini surfactants through the “click” functionalization [12].

Lin et al. ascertained the relative contributions to adsorption performance from (1) direct competition for sites and (2) pore blockage. A conceptual model was proposed to further explain the phenomena [13]. And we further explained the phenomena suggesting a promising application of cubic mesoporous BPS in wastewater treatment [5]. Hexane, octane, phenyl, and biphenyl-bridged bis (triethoxysilyl) precursors can be used in synthesizing cubic mesoporous (BPS) copolymers. The textural properties of ordered mesoporous hexane bridged polysilsesquioxane (BPS) can be tailored by choosing reaction conditions [2]. And three-dimensional cubic (Pm3n) periodic mesoporous silsequioxanes (PMS) with alkylene bridging groups was prepared by surfactant templating. They are promising candidates for organic pollutant adsorbents from water or air [3]. A vinyl silsesquioxane (VS) was added to a pesticide (citral) to enhance residual, thermal, and anti-ultraviolet properties via three double-bond reactions in the presence of an initiator [14]. After the zeolite molecular sieves were modified by polyhedral oligomeric silsesquioxane (POSS-modified ZMS), the NH_3_^+^ ino-exchange capacity increased, causing the NH_3_–N removal capacity to be enhanced [15].

The mesoporous materials with different properties and characteristics can be prepared by changing the templates and conditions of neutral long chain amines, eighteen alkyl polyoxyethylene ether, and CPBr [16,17,18,19,20]. These mesoporous materials can be widely used in catalysis, semi-conductive nanometer microcrystalline materials, adsorption, and separation fields. The ordered mesoporous silica synthesized by the surfactant template can be used in the fields of catalysis, ion exchange, adsorption, chemical sensors, and nanomaterials with much potentiality [21,22]. At the same time, different ways have also been studied in depth, such as the traditional hydrothermal method, alcohol heating method, two step sol-gel method, morphology control method, skeleton doping method, and surface modification method. Hot water synthesis can be done according to the organic groups, templates, catalysts (acid or alkali), time (agitation, aging, drying). In addition, organic-inorganic grafting was a hot topic in the research of mesoporous molecular sieve in recent years [23,24]. The ordered mesoporous materials prepared with the modified bridge SSO had the advantages of large specific surface area, adjustable pore size, narrow pore distribution, high heat resistance, and good thermal stability, and can show a simple cubic (Pm3n) mesoporous structure. The liquid-crystal-templating mechanism, the generalized liquid-crystal-templating mechanism, and other models were proposed to explain the formation of mesoporous with regular arrangement [25,26,27,28]. The selection of bridging groups helped to promote the development and application for these kinds of mesoporous cube materials, which was of great significance for the adsorption of organic pollutants, it can help to promote the development and application of these mesoporous 3D cubic materials [2,3,5,13,14,15].

Since the 1980s, it has been known that silicon dioxide extracted from rice husks has high activity and performance. Therefore, more attention has been paid to the preparation of high purity silica from rice husks. Rice husk resources are very abundant (>40 million tons/year) in China. The content of silicon in rice husk ash is relatively high, and the SiO_2_ content after high temperature calcination is 93.1%. However, most rice husks are burned as garbage, and even become the main source of pollution in some areas. The preparation of silicon organic chemicals from rice husk ash not only broadens the application scope of rice husk ash, but also solves the problem of environmental pollution. Mesoporous molecular sieve SBA-15 had many advantages, such as a large specific surface area, the pore structure rule, convenient pore size regulation, and good thermal stability. It has a broad application prospect in catalytic chemistry and adsorption separation. Therefore, this paper used rice husk as a silicon source and synthesized and studied SBA-15 and SBA-15-SH. It was hoped that the research results of this paper can be used as adsorbents to achieve the purpose of removing ammonia nitrogen from water.

## 2. Materials and Methods

### 2.1. Materials

P123 template (analytically pure) was purchased from Sigma Chemical Co. ( St. Louis, MO, USA) (3-Mercaptopropyl) trimethoxysilane (MPTMS) (industrial grade) was purchased from Nanjing Fine Chemical Co., Ltd. (NanJing, China), Anhydrous toluene and ethyl acetate (analytically pure) were purchased from Tianjin BASF Chemical Co., Ltd. (Tianjin, China), Dichloromethane (analytically pure) was purchased from Tianjin Fuyu Fine Chemical Co., Ltd. (Tianjin, China), Hydrochloric acid (37%) and anhydrous ethanol (100%) were obtained from Fisher Scientific (Waltham, MA, USA). Distilled water was obtained from the laboratory, homemade. All reagents were used as received without further purification.

### 2.2. Direct Synthesis in Hot Water

Two grams of poly (ethylene oxide)-poly (propylene oxide)-poly (ethylene oxide) block copolymer (PEO−PPO−PEO ((EO)20 (PO)70 (EO)20), namely P123) template was dissolved in 30 g of deionized water and stirred for 1 h by constant temperature magnetic stirrer bathed at 40 °C. After adding the treated rice husk ash as silicon source, it was stirred for 30 min violently. An amount of 0.4 g of MPTMS was added and stirred for 45 min. An amount of 10 mL of concentrated hydrochloric acid was added, the mixture was then stirred for 2 h continually. The solution was put in a stainless steel self-pressure autoclave lined with tetrafluoroethylene (PTFE) and then crystaled for 48 h in an oven at 100 °C. It was then poured out and a filtration was taken, it was washed two times repeatedly with 100 mL of ethanol /hydrochloric acid (50:1 *v*/*v*) solution for 4 h, the template P123 was removed and then the directly synthesized thiol modified mesoporous SBA-15 (H-SBA-15SH) was obtained. The experiment was performed in triplicate. IR (KBr pellet): ν = 3434, 1608, 1078, 956, 807, 456 cm^−1^; ^13^C CP MAS-NMR (50.20 MHz) δ = 49.5 (SiCH_2_CH_2_C^3^H_2_SH), 27.4 (SiCH_2_C^2^H_2_CH_2_SH), 9.1 (SiC^1^H_2_CH_2_CH_2_SH); 29Si CP MAS-NMR (39.65 MHz) δ = −48.85 ((SiO)–Si–(OH)_2_R, T^1^ cyclic), −57.11 ((SiO)_2_–Si–(OH) R, T^2^ cyclic), −68.31 ((SiO)_3_–Si–R, T^3^ cyclic), −101.03 ((SiO)_3_Si–(OH), Q^3^ silicon atoms), −110.35 ((SiO)_4_Si, Q^4^ silicon atoms).

### 2.3. Grafting Synthesis

Two grams of P123 was weighed and dissolved in 30 g of deionized water, stirred for 2 h, bathed at 40 °C to dissolve it, 12.63 g of concentrated hydrochloric acid was added, and it continued to be stirred for 0.5 h. The treated rice husk ash was added and stirred for 3 h. The solution was put into a 50 mL reaction vessel, and crystallized in an oven at 100 °C for 72 h. The mixture was washed with distilled water and filtered, after drying the original powder of SBA-15 sample. Then the sample was heated to 550 °C at a speed of 2 °C/min in air atmosphere and calcined at this temperature for 6 h to remove the template, namely the mesoporous molecular sieves SBA-15. IR (KBr pellet): ν = 1642, 1090, 962, 810, 469 cm^−1^; ^13^C CP MAS-NMR (50.20 MHz) δ = 76.7 (SiCH_2_CH_2_C^3^H_2_OH), 73.9 (SiCH_2_C^2^H_2_CH_2_OH), 18.9 (SiC^1^H_2_CH_2_CH_2_OH); ^29^Si CP MAS-NMR (39.65 MHz) δ = −91.74 ((SiO)_2_Si–(OH)_2,_ Q^2^ silicon atoms), −98.72 ((SiO)_3_Si-(OH)_,_ Q^3^ silicon atoms), −109.87 ((SiO)_4_Si_,_ Q^4^ silicon atoms).

One gram of SBA-15 and 0.36 g of MPTMS were dissolved in 100 g of anhydrous toluene, stirred, refluxed for 24 h, and then filtered. The filtered solid was refluxed and washed for 24 h with a solution containing 50 g of dichloromethane and 50 g of ethyl acetate. After removing the MPTMS from the surface of the sample, a mercapto group-containing surface modified molecular sieve SBA-15 was obtained, namely G-SBA-15-SH. IR (KBr pellet): ν = 1092, 780, 600, 546, 450 cm^−1^; ^13^C CP MAS-NMR (50.20 MHz) δ = 49.1 (SiCH_2_CH_2_C^3^H_2_SH), 27.3 (SiCH_2_C^2^H_2_CH _2_SH), 9.6 (SiC^1^H_2_CH_2_CH_2_SH); ^29^Si CP MAS-NMR (39.65 MHz) δ = −49.21 ((SiO)–Si–(OH)2R, T^1^ cyclic), −58.24 ((SiO)_2_–Si–(OH)R, T^2^ cyclic), −69.56 ((SiO)_3_–Si–R,T^3^ cyclic), −102.17 ((SiO)_3_Si–(OH), Q^3^ silicon atoms), −111.20 ((SiO)_4_Si, Q^4^ silicon atoms).(You can see the details in the Supplementary).

### 2.4. Structure Characterization

X-ray diffraction analysis (XRD): A Philips X’ pert instrument A LabX XRD-6000 X-ray diffractometer (Shimadzu Corporation, Kyoto, Japan) using monochromatic Cu Kα radiation was used to record powder X-ray diffraction patterns. A short wavelength particle or a crystalline material was used to reflect the photon surface of an atomic force plane, thus forming a three-dimensional structural form at the atomic force level [29]. Test conditions: CuKa line, λ = 0.15418 nm, tube voltage was 40 kV, tube current was 30 mA, 1°/min of scanning speed.

Fourier Transform Infrared spectrogram (FTIR): Thermo Nicolet Avatar 360 Fourier Transform Infrared Spectrometer (Thermo Fisher Scientific, Waltham, MA, USA) was used to characterize dried samples.

Scanning electron microscopy (SEM): A US-made Quanta 200F electron microscope (FEI, Hillsboro, OR, USA) test sample with an accelerating voltage of 29 KV was used. There was a sample test before the spray treatment.

Transmission electron microscopy (TEM): A Hitachi H-8100 instrument (Hitachi, Tokyo, Japan) at an accelerating voltage of 200 kV recorded the TEM images. The grinded sample was put into the weighing bottle with ethanol as a dispersant and then shocked for 5 min by ultrasonic. After that, the drop solution was taken by a clean drip tube onto the copper, the copper was dried, and the particle morphology of the sample was observed on the machine.

Proton-decoupled ^13^C and ^29^Si solid-state NMR spectra were recorded on a Bruker DSX Avance spectrometer. 

Nitrogen adsorption–desorption: the nitrogen adsorption–desorption isotherms of the samples were measured at −196 °C and the samples were degassed at 150 °C for 8 h under vacuum. The specific surface area of the sample was calculated by the Brunauer–Emmett–Teller (BET) equation, and the pore size distribution curve was calculated by the Barrett–Joyner–Halenda (BJH) method [30].

The BJH method based on the Kelvin formula has a thermodynamic origin. In the BJH method, the mesopore pore size distribution is usually expressed by graphical form △*V*_p_/△*r*_p_ & *r*_p_ or *d*_p_ (*V*_p_ is the volume of mesopore, *r*_p_ is the radius of the cylindrical hole, and *d*_p_ is the width of the parallel to crack inside). The mesopore volume is completely filled at a relatively high pressure. The pore size distribution depends on whether there is a hysteresis loop. However, the new BJH method is more emphasis on Density Functional Theory [31,32,33] and Monte Carlo simulation [34]. MCM-41 molecular sieve materials characterized by Density Functional Theory has been reported but used rarely [35].

BET equations are used to determine the specific surface area of mesoporous solids [36]. Under relative low-pressure conditions, the BET equation is the abbreviation of the Langmuir equation and it is relatively good to describe the adsorption process under the condition of relative pressure 0.05–0.35. This specific surface area, as (BET), was obtained from the following equation.

$$a_{s}\;\left(BET\right)=n_{m}^{a}\times L\times a_{m}$$

*L* is the avogadro constant, $n_{m}^{a}$ is the amount of adsorption of a single coating (the surface of the adsorbent in the unit to form a complete single coating), $a_{m}$ is the average area that accounts for the proportion of adsorbate molecules.

### 2.5. Effect of the Initial Concentration of Ammonia Nitrogen on the Adsorption of Ammonia Nitrogen

Every modified zeolite (modified by sodium chloride) of a molecular sieve of SBA-15, H-SBA-15-S, G-SBA-15-SH respectively weighed exactly 0.1 g. The number of every weighed molecular sieve should meet the requirements of the following NH_4_Cl standard solution concentration gradient. Then every one of them was added into a 50 mL triangular flask, at the same time, 50 mL NH_4_Cl standard working solution with a concentration of 0.1, 0.2, 0.4, 0.8, 1.6 and 2 mg/L were respectively used to test. The solutions were oscillated 120 min by the air flow oscillator, and then static for a period of time, filtering with microporous filter membrane, after that the supernatant was determined by spectrophotometry. Calculating the concentration of ammonia nitrogen in a solution by a standard curve, the test results were as shown in Table 1. The concentration of residual ammonia nitrogen after treatment with three kinds of molecular sieves was calculated by the following formula:

$$q\left(mg/g\right)=\;\left(C_{0}-C\right)\times V/G$$

*q* is the adsorption quantity of ammonia nitrogen by zeolite, $C_{0}$ is the concentration of ammonia nitrogen in the simulated raw water (mg/L), C is the concentration of ammonia nitrogen in solution after treatment (mg/L), *V* is the volume of the added NH_4_Cl simulated solution (ML), *G* is the mass of zeolite (g).

### 2.6. Statistical Analysis

All experiments were performed in triplicate, as the replicated experimental units and the results were provided with mean ± SD (standard deviation) values. One-way analysis of variance (ANOVA) was performed, and the significance of each mean value was determined (*p* < 0.05) with the Duncan’s multiple range test of the statistical analysis system using the SPSS computer program (SPSS, Inc., Chicago, IL, USA).

## 3. Results and Discussion

### 3.1. X-Ray Diffraction

**Figure 1:**
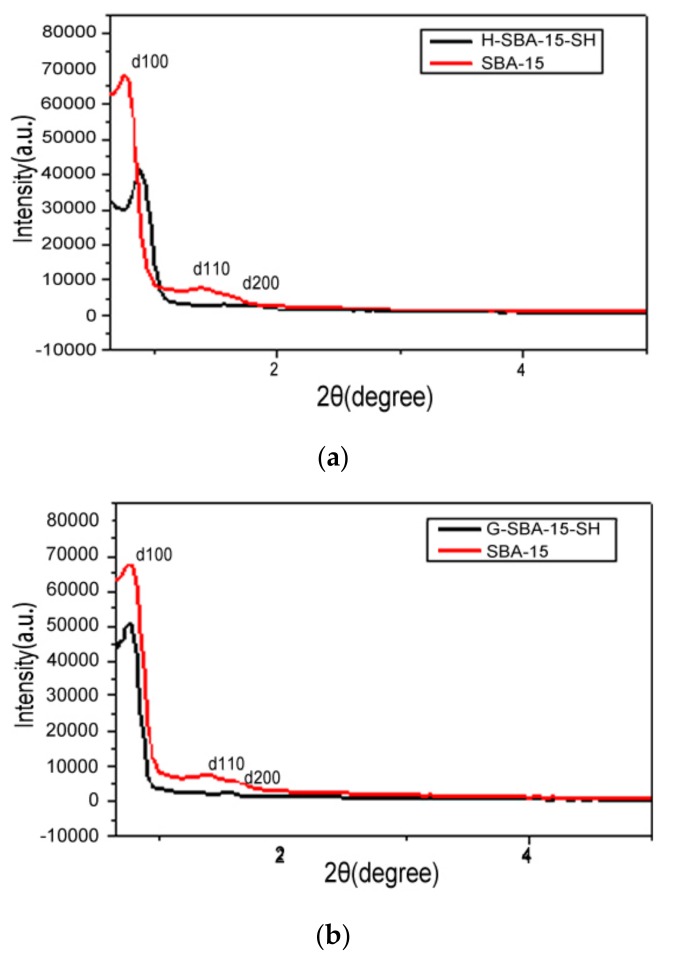
(**a**) X-ray diffraction patterns of mesoporous SBA-15 and H-SBA-15-SH; (**b**) X-ray diffraction patterns of mesoporous SBA-15 and G-SBA-15-SH.

According to the Prague formula (*n*λ = 2*d*sinθ, d is the distance of the atomic crystal plane in the crystal phase, *n* is an integer, and θ is the Bragg angle), the sample was diffracted in the crystalline phase. The intensity of the X-ray diffraction depends on the 2θ angle diffraction function and the crystal orientation of the sample. The diffraction properties determine the structural properties of the sample (e.g., crystal orientation and size) and the crystalline phase. The orderliness of the pore material was measured by X-ray diffraction with powder. Ordered mesoporous organosilicon material has a very high diffraction peak when 2θ = 2° and a low diffraction peak in the range of 2θ varied from 3° to 8° [23,24]. The cell parameters are calculated by the formula a_0_ = 2*d*_100_/ (3)^1/2^ in the hexagonal mesopore phase [23]. The difference of the cell parameters and the pore size (measured by nitrogen adsorption–desorption) gives the mesoporous phase pore-thickness. Small angle X-ray diffraction can determine the mesoporous phase of ordered mesoporous materials. A strong diffraction peak d_100_ appears at the diffraction angle 2θ of 0.80° and another two weak diffraction peaks *d*_100_ and *d*_200_ appear at 1.4°–1.8°, as shown in Figure 1a, b. The black line represents SBA-15-SH and the red line represents the SBA-15. The three diffraction peaks are typical characteristic peaks of two-dimensional hexagonal structures, and these are attributed to the (100), (110), (200) crystal diffraction peaks of the two-dimensional hexagonal (p6mm) structure respectively, and the d values of the diffraction peaks of different crystal planes satisfy the characteristic relation of hexagonal lattice.

The XRD patterns of the two mesoporous molecular sieves of SBA-15 and H-SBA-15-SH are illustrated schematically in Figure 1a, the three absorption peaks of the chromatogram of H-SBA-15-SH synthesized by direct synthesis method, corresponding to facet absorption peaks of SBA-15 (100, 110, 200) respectively. The intensity of the three absorption peaks is significantly weaker than that of SBA-15, and not only are the main peak of *d*_110_ and characteristic peaks of *d*_200_ and *d*_100_ significantly weakened, but also the *d*_100_ peak of H-SBA-15-SH is drifted at a high angle compared with *d*100 peak of SBA-15, which related to thiol getting into the molecular sieve skeleton.

The XRD patterns of the two mesoporous molecular sieves of SBA-15 and G-SBA-15-SH are illustrated schematically in Figure 1b. The three absorption peaks of the chromatogram of H-SBA-15-SH synthesized by the post-grafting method, also correspond to facet absorption peaks of SBA-15 (100, 110, 200) respectively. Unlike H-SBA-15-SH, the intensity of these peaks hardly changes, and the peak of d100 of G-SBA-15-SH almost has no drift compared with that of SBA-15, which indicates that no effect is produced after introducing the mercapto group into the molecular sieve framework by the post-grafting method, and the G-SBA-15-SH maintain a good pore structure and regular pore arrangement. In a word, the ordered mesoporous materials of G-SBA-15-SH and the synthesized H-SBA-15-SH are consistent with those reports [24].

### 3.2. Fourier Transform Infrared Spectroscopy

SBA-15 shows the characteristic bands at 1642, 1090, 810, and 469 cm^−1^, which were attributed to the O–H vibration of the silanol (Si–OH) group or the silanol group of the cross-hydrogen bond [37,38], the symmetric stretching vibration of the Si–OH swing mode [37], the Si–O–Si asymmetric stretching vibration [37,39], the Si–O–Si symmetric vibration [39], and the bending mode of Si–O–Si [40].

At the peak of 1078 cm^−1^, the peak of H-SBA-15-SH had a tendency to move to the right with respect to the SBA-15, which was caused by the asymmetric Tensile vibration of Si–O–Si [40], while the peak of G-SBA-15-SH was not quite different from the location of SBA-15. It could be found that there was a sharp band between H-SBA-15-SH and G-SBA-15-SH at 3000–2800 cm^−1^, which was attributed to the C–H stretching belt of propyl group [40].

### 3.3. Scanning Electron Microscopy

**Figure 2:**
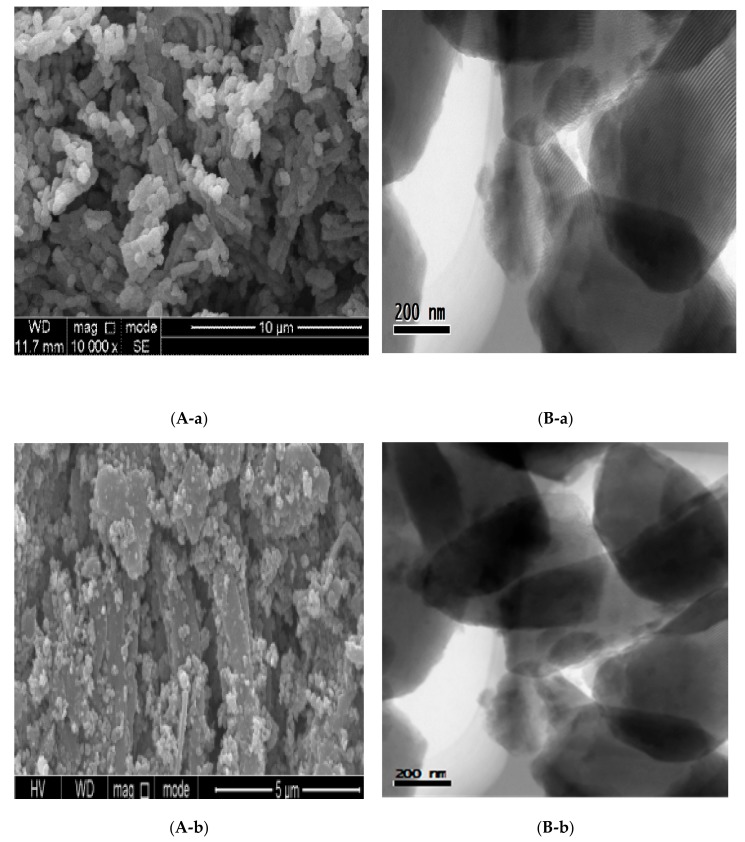

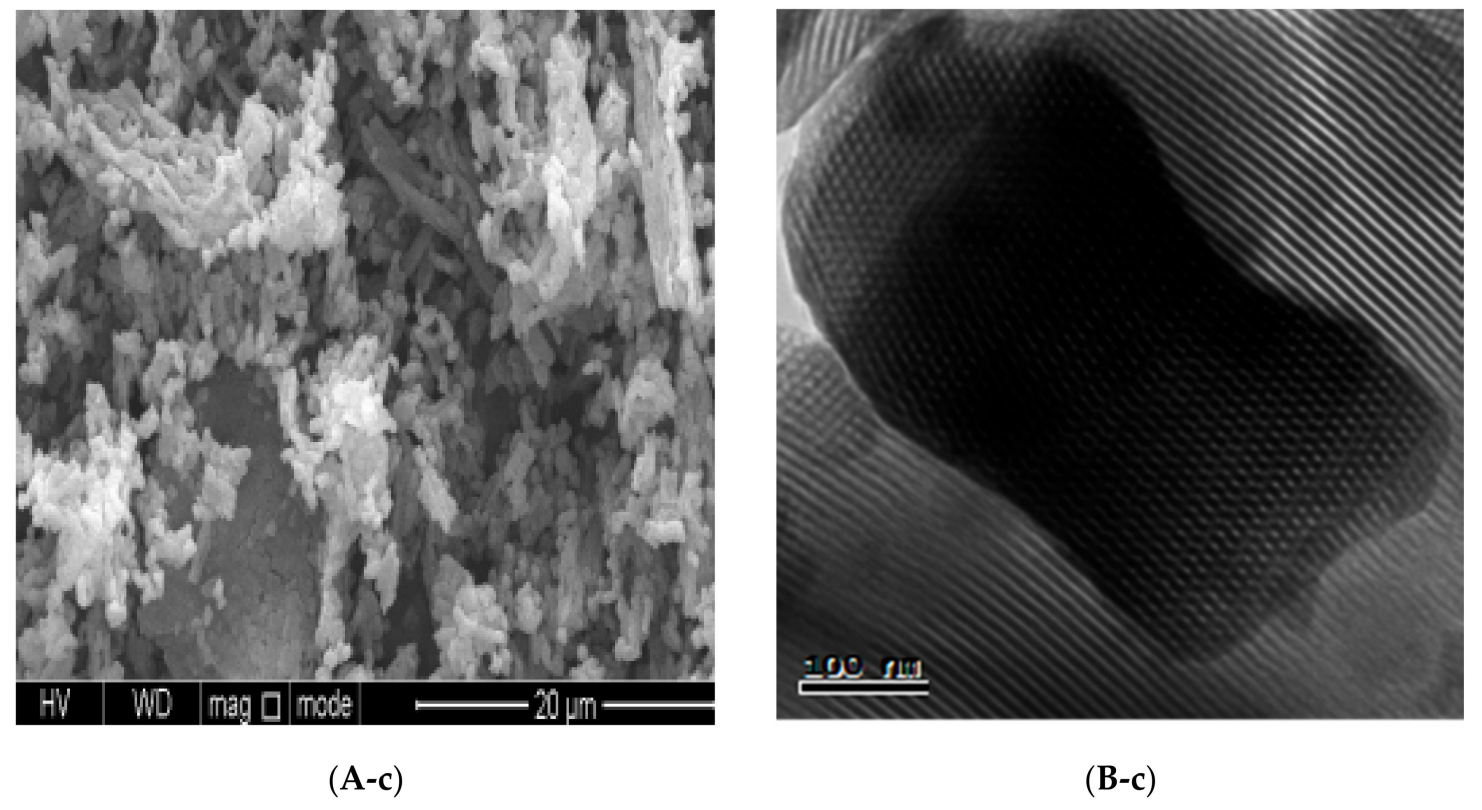
Scanning electron microscopy (SEM (**A**) and transmission electron microscopy (TEM) (**B**) of mesoporous SBA-15 (**a**); H-SBA-15-SH (**b**); G-SBA-15-SH (**c**).

Scanning electron microscope (SEM) photos of SBA-15 and SBA-15-SH mesoporous molecular sieves were shown in Figure 2A. It can be seen that the structure is evenly distributed [41]. The SBA-15 mesoporous molecular sieve had a short cylindrical appearance. After the incorporation of the functional group, it still maintained the hexangular mesoporous structure of SBA-15 [42], but the shape of H-SBA-15-SH was more dispersed and small, and the shape of G-SBA-15-SH was slightly longer. The obvious surface roughness on SEM images may be due to the presence of organic functional groups on the surface and the presence of MPTMS [43]. Overall, the mesoporous structure of SBA-15 was greatly retained in organic functionalization, which was consistent with the XRD results [44].

### 3.4. Transmission Electron Microscopy

The internal morphology of SBA-15 and SBA-15-SH mesoporous molecular sieves were studied by TEM as shown in Figure 2B. Figure 2B showed a better honeycomb structure in the middle of the picture [45]. In Figure 2B-a, it clearly indicated the formation of SBA-15 with a highly ordered hexangular mesoporous structure, where hexangular channels were clearly observed [46]. The SBA-15’s tunnel was parallel to the axis and was arranged in an orderly regular cylindrical channel in the direction perpendicular to the axis [46], thus further proving that SBA-15 had a two-dimensional p6mm hexangular structure. After the synthesis of SBA-15-SH, the structure of SBA-15 remained to be maintained, as shown in Figure 2B-b,B-c. The addition of (3-mercapto propyl) trimethoxane did not damage the porous structure of the six party [46].

### 3.5. Analysis of Pore Size Distribution

**Figure 3:**
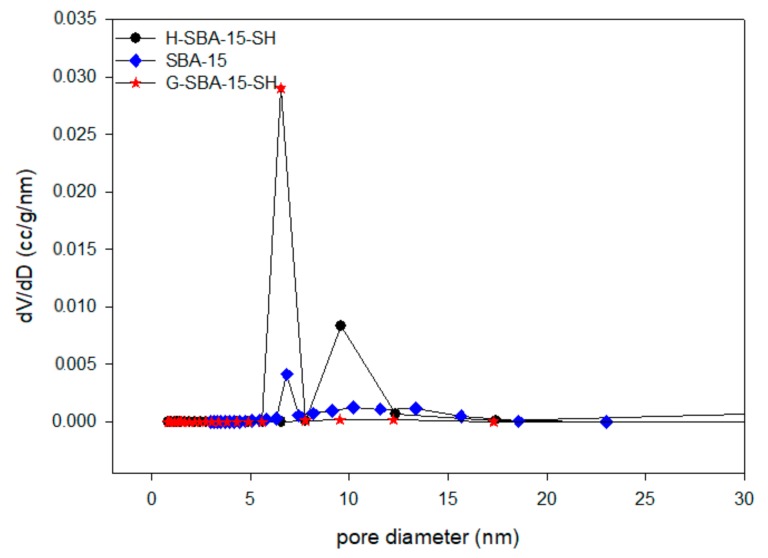
Pore size distributions of mesoporous SBA-15, H-SBA-15-SH, G-SBA-15-SH.

Figure 3 shows that the pore size distribution curve of mesoporous molecular sieves SBA-15, which is a typical mesopore pore size distribution with a narrow pore size distribution, varied from 5 to 7 nm (Figure 3). The average pore size was 6.85 nm calculated by the new BJH method. When nitrogen was at 77 K·am, (N_2_) = 0.162 nm^2^. The specific surface area of the mesoporous SBA-15 was 310.1 m^2^/g measured by the BET formula, and the pore volume was 0.43 cm^3^/g. However, the BET was lower than that of mesoporous silicone reported in the literature (greater than 1000 m^2^/g) [47].

The pore–size distribution curves of mesoporous SBA-15, H-SBA-15-SH and G-SBA-15-SH are shown in Figure 3. As shown in Figure 3, mesoporous H-SBA-15-SH synthesized by direct synthesis and mesoporous G-SBA-15-SH synthesized by post-grafting synthesis both had homogeneous mesoporous structures, but the pore size distribution showed different changes. The pore size distribution of mesoporous H-SBA-15-SH was wider than that of SBA-15. It showed that direct synthesis was a direct introduction of a small amount of mercapto into the skeletal structure of SBA-15, causing pore diameter to increase. The pore size distribution of mesoporous G-SBA-15-SH was narrower than that of mesoporous SBA-15, which indicated that post-grafting method combined a large amount of mercapto groups with siloxanes on the surface of SBA-15 and entered into the inner pores so that the thickness of the hole wall increased and pore size reduced. The conclusion is that the pore size distribution curve was consistent with the XRD analysis.

### 3.6. Analysis of Nitrogen Adsorption–Desorption

**Figure 4:**
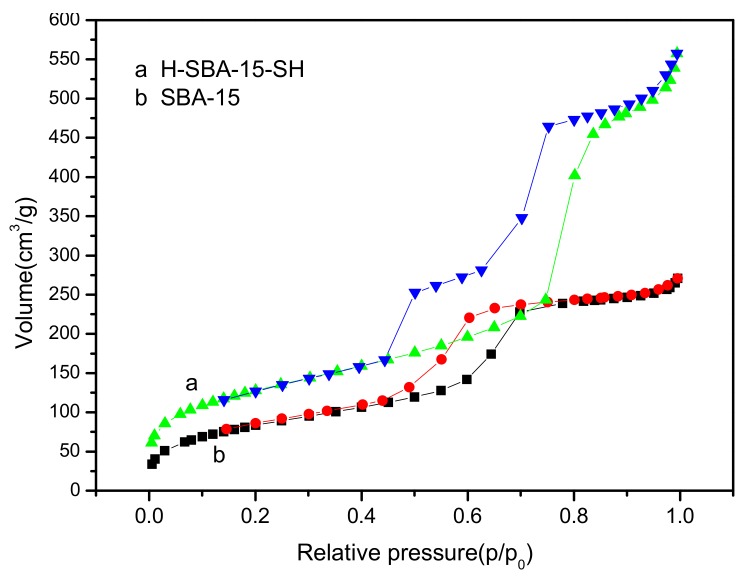
N_2_ adsorption isotherms of mesoporous SBA-15 and H-SBA-15-SH.

The N_2_ adsorption–desorption isotherm of the mesoporous molecular sieves SBA-15 is shown in Figure 4b. It can be seen that the N_2_ adsorption–desorption isotherms drawn according to the results of the test conform to the typical characteristics of the adsorption isotherm type IV set by IUPAC [36], and meanwhile it showed that the synthetic materials had a mesoporous structure. At the lower relative pressure, the monolayer adsorption occurred, the curves of which showed that the adsorption and desorption lines coincided. Then, multilayer adsorption occurred until the pressure was sufficient to cause capillary condensation. The adsorption isotherm changed greatly, when the adsorption hysteresis occurred, namely the adsorption and desorption curves were separated from each other. In this case, the isotherm had a hysteresis loop. There was an adsorption hysteresis in Type IV due to capillary condensation and a relatively high relative pressure (0.45 < $p/p_{0}$ < 0.8). The hysteresis loop of type H1 [36,48] existed with parallel and close vertical branches as shown in Figure 4. Type H1 represented adsorption in a narrow mesopore with a small pore size distribution, which was mostly caused by the uniform size and regular shape of the hole. In the range of the small relative pressure, the curve was relatively flat, which may be because nitrogen molecules were adsorbed on the surface of the mesoporous. In the range of the high relative pressure, the adsorption capacity increased rapidly with the increase in relative pressure, which may be related to the nitrogen molecules adsorbed to the mesoporous pores by the monolayer and multilayer adsorption, causing capillary agglomeration. Then the adsorption capacity increased slowly with the increase in the relative pressure and the adsorption gradually saturated. At the same time, there was a large pressure in the middle pressure zone, which was related to the larger pore size of mesoporous molecular sieve SBA-15.

**Figure 5:**
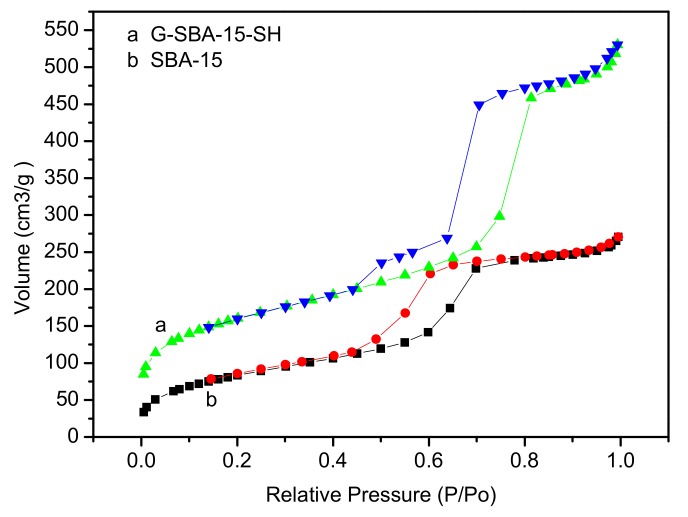
N_2_ adsorption isotherms of mesoporous SBA-15 and G-SBA-15-SH.

N_2_ adsorption isotherms of mesoporous are as shown in Figure 4 and Figure 5. From Figure 4a, the H-SBA-15-SH synthesized by the direct synthesis method has the type IV of adsorption–desorption curve [36,48]. The hysteresis loop shape was similar to that of SBA-15. And with the increase in relative pressure ($p/p_{0}$ > 0.42), the adsorption curve also has an obvious breakthrough, which was typical capillary adsorption of mesoporous with uniform pore distribution. But unlike Figure 4b, N_2_ adsorption capacity increased as shown in Table 1, the pore size increased from 6.85 to 9.26 nm, the specific surface area increased from 310.1 to 571.3 m^2^/g, and the pore volume increased from 0.43 to 0.88 cm^3^/g. Thus, the H-SBA-15-SH synthesized by direct synthesis has a larger pore size and specific surface area and can further synthesize the carrier of mesoporous catalyst.

The N_2_ adsorption–desorption isotherm of mesoporous G-SBA-15-SH synthesized by post-grafting was shown in Figure 5a, the isotherm also had type IV of the adsorption–desorption curve [36,48]. The hysteresis loop shape was also similar to that of SBA-15 ordered mesoporous molecular sieves. With the increase in relative pressure ($p/p_{0}$ > 0.42), the adsorption curve had an obvious breakthrough, which was also a typical capillary adsorption agglomeration of mesoporous with a uniform pore distribution [36,48]. But there was some difference from N_2_ adsorption isotherms in ordered mesoporous molecular sieve H-SBA-15-SH synthesized by the direct synthesis method. The hysteresis loop synthesized by the post-grafting method was elongated compared with the SBA-15 ordered mesoporous molecular sieves, the N_2_ adsorption capacity decreased and the hysteresis loop shifted to the high $p/p_{0}$ value. The hysteresis loop may change from H4 to H3.

The amount of ammonia nitrogen adsorbed was measured by the mass of ammonia nitrogen in water removed by molecular sieves per unit mass. The relationship between the amount of ammonia nitrogen adsorption and the initial concentration of ammonia nitrogen was shown in Table 2, Table 3 and Table 4. With the increase in the concentration of ammonia and nitrogen, the adsorption amount of ammonia nitrogen increases gradually. That was, with the increase in the ammonia nitrogen initial concentration, the adsorption capacity of the modified molecular sieve to ammonia nitrogen was constantly improved. At the same time, it can be found that the adsorption amount of ammonia nitrogen by G-SBA-15-SH molecular sieve was the largest, the H-SBA-15-SH molecular sieve was the second, while the unmodified SBA-15 molecular sieve had the smallest ammonia nitrogen adsorption. Besides, with the increase in ammonia nitrogen concentration, the ammonia nitrogen adsorption capacity changed more and more. When the concentration was very low, it was difficult to adsorb.

## 4. Conclusions

In acidic conditions, pure silicon synthetic SBA-15 with P123 as the template and rice husk ash as the silica source was prepared and was analyzed by FTIR, XRD, SEM, TEM, ^13^C CP MAS-NMR, ^29^Si CP MAS-NMR and BET. The result showed that synthetic sample mesoporous SBA-15 had a two-dimensional hexagonal structure with an average pore size of 6.85 nm. The specific surface area of the mesoporous SBA-15 was 310.1 m^2^/g and the pore volume was 0.43 cm^3^/g. The direct synthesis method of the ordered mesoporous molecular sieves H-SBA-15-SH is the way of introducing the mercapto directly, causing both the pore size, the pore volume, and the specific surface area to increase. The mesoporous molecular sieve can further synthesize the mesoporous catalyst carrier. The ordered mesoporous molecular sieves G-SBA-15-SH synthesized by post-grafting proved that the sulfhydryl group was bonded to the silyl group on the surface of the G-SBA-15-SH, and combined into the interior of the channel, causing the hole wall thickness to increase and the pore size to reduce. The N_2_ adsorption capacity decreased. According to the results of the experiment, the ammonia-nitrogen from water can be expected to be removed by the SBA-15-SH.

## Figures and Tables

**Table 1:** Textural properties of mesoporous zeolites.

Molecular Sieve Samples	Specific Surface Area (m^2^/g)	Pore Volume (cm^3^/g)	Average Pore Size (nm)
SBA-15	310.1	0.43	6.85
H-SBA-15-SH	571.3	0.88	9.26
G-SBA-15-SH	463.8	0.85	6.53

**Table 2:** Removal of ammonia nitrogen from SBA-15 molecular sieve.

Concentration of Ammonia Nitrogen Solution (mg/L)	Ammonia Nitrogen Content in 50 mL Solution (mg)	The Content of Ammonia Nitrogen in the Solution after Adsorption (mg)	The Additive Amount of Molecular Sieve (g)	Ammonia Nitrogen Adsorption Capacity (mg/g)
0.1	0.005	0.0048	0.1	1.667
0.2	0.01	0.0096	0.1	3.667
0.4	0.02	0.0193	0.1	7.444
0.8	0.04	0.0387	0.1	13.22
1.6	0.08	0.0759	0.1	21.00
2	0.1	0.0971	0.1	29.00

**Table 3:** Removal of ammonia nitrogen from H-SBA-15-SH molecular sieve.

Concentration of Ammonia Nitrogen Solution (mg/L)	Ammonia Nitrogen Content In 50 mL Solution (mg)	The Content of Ammonia Nitrogen in the Solution after Adsorption (mg)	The Additive Amount of Molecular Sieve (g)	Ammonia Nitrogen Adsorption Capacity (mg/g)
0.1	0.005	0.0050	0.1	2.551
0.2	0.01	0.0096	0.1	6.332
0.4	0.02	0.0198	0.1	11.04
0.8	0.04	0.0391	0.1	17.01
1.6	0.08	0.0796	0.1	24.72
2	0.1	0.0997	0.1	34.33

**Table 4:** Removal of ammonia nitrogen from G-SBA-15-SH molecular sieve.

Concentration of Ammonia Nitrogen Solution (mg/L)	Ammonia Nitrogen Content in 50 mL Solution (mg)	The Content of Ammonia Nitrogen in the Solution after Adsorption (mg)	The Additive Amount of Molecular Sieve (g)	Ammonia Nitrogen Adsorption Capacity (mg/g)
0.1	0.005	0.0050	0.1	3.312
0.2	0.01	0.0096	0.1	7.898
0.4	0.02	0.0120	0.1	14.17
0.8	0.04	0.0390	0.1	19. 02
1.6	0.08	0.0781	0.1	30. 32
2	0.1	0.0970	0.1	41.31

## Supplementary Materials

The following are available online at www.mdpi.com/link.
