# Supplementary figures and images for: Preparation and Characterization of Highly Ordered Mercapto-Modified Bridged Silsesquioxane for Removing Ammonia-Nitrogen from Water
Source: Polymers (Basel). 2018 Jul 25;10(8):819. doi: 10.3390/polym10080819 (PMC6403600; doi:10.3390/polym10080819)

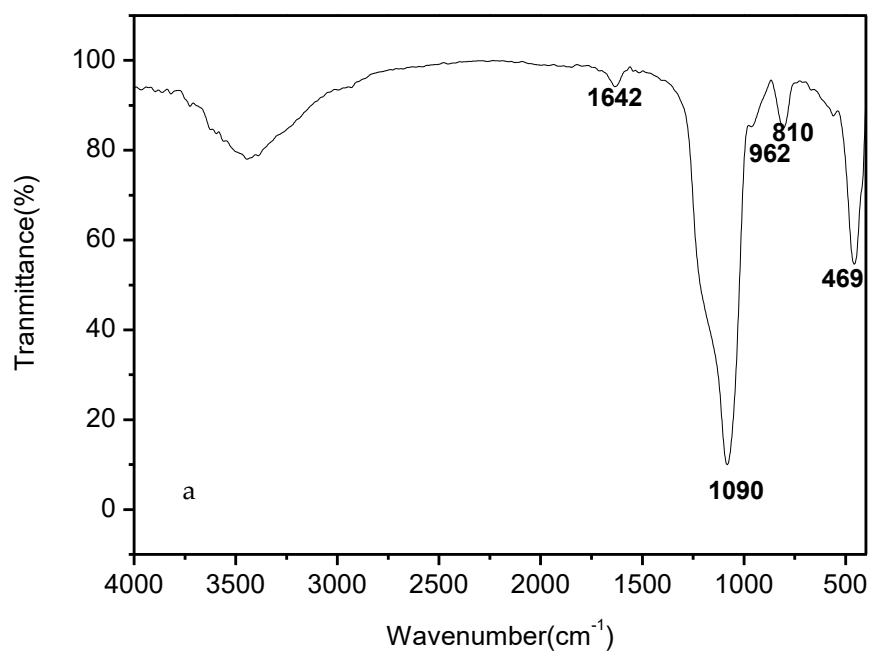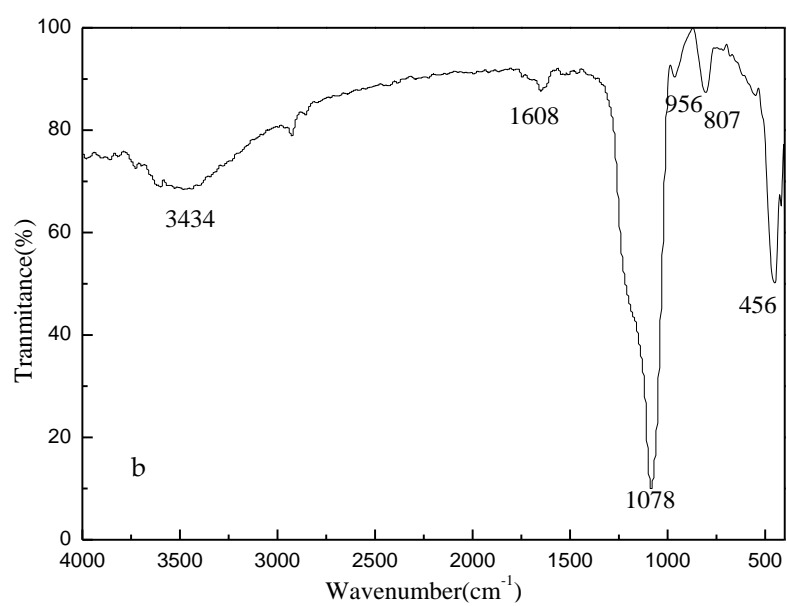

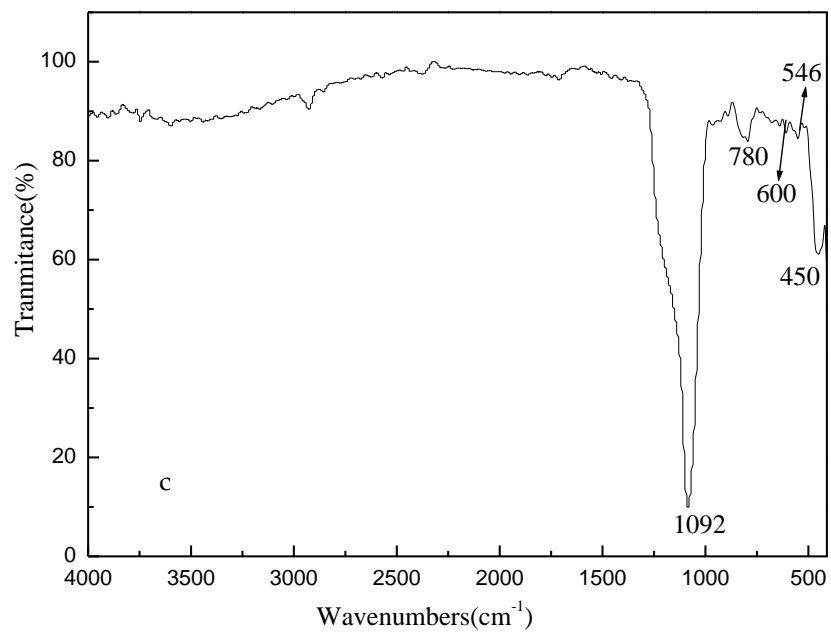

Fig. S1 FTIR spectra of mesoporous SBA-15 (a); H-SBA-15-SH (b); G-SBA-15-SH (c)

Supplement: Supplementary file 1 [file polymers-10-00819-s001.pdf]
